# GBIQ: a non-arbitrary, non-biased method for quantification of fluorescent images

**DOI:** 10.1038/srep26454

**Published:** 2016-05-23

**Authors:** Youichirou Ninomiya, Wei Zhao, Yumiko Saga

**Affiliations:** 1 Mammalian Development Laboratory, National Institute of Genetics, Yata 1111, Mishima, Shizuoka 411-8540, Japan

## Abstract

Non-arbitrary and non-biased quantification of fluorescent images is an essential tool for the data-centric approach to biological systems. Typical application is high-content analysis, where various phenotypic changes in cellular components and/or morphology are measured from fluorescent image data. A standard protocol to detect cellular phenotypes is cell-segmentation, in which boundaries of cellular components, such as cell nucleus and plasma membrane, are first identified to define cell segments, then acquiring various phenotypic data of each segment. To achieve reliable outcome, cell-segmentation requires manual adjustments of many parameters; this requirement could hamper automated image processing in high-throughput workflow, whose quantification must be non-arbitrary and non-biased. As a practical alternative to the segmentation-based method, we developed GBIQ (Grid Based Image Quantification), which allows comparison of cellular information without identification of single cells. GBIQ divides an image with tiles of fixed size grids and records statistics of the grids with their location coordinates, minimizing arbitrary intervenes. GBIQ requires only one parameter (size of grid) to be set; nonetheless it robustly produces results suitable for further statistical evaluation. The simplicity of GBIQ allows it to be readily implemented in an automated high-throughput image analysis workflow.

Fluorescent cytometry is an indispensable method to obtain quantitative data from fluorescent staining. For tissue sections and substrate-attach cultured cells that should be analyzed *in situ*, a combination of fluorescent microscopy and image analysis is a standard solution for obtaining reliable quantitative data from them. A starting process of the image analysis would be identification of each cell by cell-segmentation using nuclear and/or cytoplasmic counterstaining, the process eventually defines quality of the outcome. Thus, once the segmentation was established, fluorescent intensities of other molecular entities in question within the cell are measured and stored at cellular basis for further phenotypic analysis^1^. The segmentation process, therefore, should be carried out with a great deal of attention to reach optimal cell-segmentation while eliminate unwanted artifacts. This means that there are a plenty of rooms of arbitration and bias at the starting process, and requirement of fine-tunings and human interventions on image-to-image basis during the process, which are potentially time-consuming, tedious and often leading to poor reproducibility of the outcomes. These drawbacks of the cell-segmentation method would also potentially hamper a streamlined implementation of the image measurement method with an automated high-throughput data analysis workflow. In fact, many cell-segmentation methods have been developed, none of them is applicable universally, algorithms and parameter-sets should be tailored for subject specific manner^2^.

In our daily research activities at a bench, we often encounter a situation where we need quick quantitative assessment of fluorescent images that were produced by initial experiments. Even though cell-segmentation methods are robust and reliable, they are quite unsuitable in the situation due to methods’ demand for parameter settings. Especially, if the experimental subjects are densely packed cells including colonies of embryonic stem cells (ESCs) or condensing mesenchymal cells in tissue sections, it would be even more hard applying the cell-segmentation methods onto the subjects. Unlike mono-layered culturing cells, these subjects often contain complex cellular phenotypical heterogeneity and architectural diversity in a context of high-density of cells. This nature of subjects makes the cell-segmentation difficult without the tailored algorithm and a great deal of fine-tunings to the set of parameters. These arbitrary procedures inevitably take our labour and time, hence in this situation, a quick, easy and ideally parameter-free quantification method is in great demand.

A solution to the problem would be a non-arbitrary and non-biased quantification method that processes fluorescent images under minimum set of rules. One possible way to attain this could be abandoning the cell-segmentation from the process. GBIQ (stands for Grid Based Image Quantification) is designed for reducing human intervenes as little as possible to achieve both reproducibility of data and streamlined workflow. Instead of cell identification by the cell-segmentation, GBIQ employs a tiling of fixed size grids and utilizes statistics within and among the grids to quantify fluorescent images. Although GBIQ does not identify single cells in principle, the method yet produces quantitative dataset that can be classified to sub-populations among which phenotypic features vary in a subject image dataset. Here we report the process of GBIQ in comparison to the cell-segmentation based method, verifying GBIQ is a practical alternative to the segmentation-based method. GBIQ particularly well performs in cases the cell density of subject is high, where the segmentation method would require elaborative fine-tunings of parameter-sets to identify each cell, as exampled here applying the both methods on same image datasets obtained from dense colonies of mouse ESCs (mESCs). We also apply GBIQ on various tissue sections from developing mouse embryos to elucidate a gene regulatory network.

## Materials and Methods

### Cell culture

Doxycycline (Dox) inducible transgene vector (pPBhCMV-1cHApa) carrying Flag-tagged mouse Nanos2 connected with EGFP via t2a sequence was transfected into mESCs with pPBCAG-rtTAM2-IN via piggyback-based method to establish a stable transformant (PB-EGFP-Nanos2). The mESCs were cultured on feeder cells derived from mouse embryonic fibroblasts treated with Mitomycin C. For immunostaining, the feeder cells were prepared on gelatin-coated coverslips then PB-EGFP-Nanos2 cells were inoculated and cultured on the feeder cells for few days. To induce transgenes, Dox dissolved dimethyl sulfoxide (DMSO) was added for 24 hours at final concentration of 1 μM Dox. Same amount of DMSO was also added as the vehicle control.

### Immunofluorescent staining

MESCs on coverslips were fixed with 4% paraformaldehyde in Ca, Mg-free phosphate buffered saline (PBS(−)) for 30 minutes on ice then rinsed several times with PBS(−). These samples were stored for up to a month at 4 °C in dark. Indirect immunofluorescent staining was performed at room temperature in dark and using 0.1% Tween20 supplemented PBS(−) (PBT) unless otherwise stated. Prior to apply primary antibody, these samples were incubated with 0.1 mg/ml bovine serum albumin and 10% heat inactivated fetal calf serum supplemented PBT (blocking solution) for 2 hours. Anti-Oct4 (Pou5f1) (Santa Cruz, sc-5279, 1:100) with indicated dilution ratio in the blocking solution was applied for overnight at 4 °C. To visualize the immunoreactions and cell nuclei, AlexaFluor 594 conjugated anti-mouse IgG(H+L) raised by donkey (Molecular Probes, diluted 1:500 in PBT supplemented with 1 μg/ml H33342) were applied for 2 hours at room temperature in dark. Samples were mounted on slide glasses with 80% glycerol in PBS(−) supplemented with 1 μg/ml H33342 and subjected to image acquisition using laser scanning confocal microscopy (FV-1200, Olympus). Triple (Tbx6, Mesp2 and Ripply2) immunofluorescent staining of developing tailbud from mouse embryos was carried out in accordance with previous report^3^.

### Image quantification and statistical analysis

Cell-segmentation and consequent fluorescent cytometry was carried out using TissueQuest 4.0 (TissueGnostics, hereafter TQ). Nuclear counterstaining was used as master image for cell identification with following parameter set.

Nuclei Size: 20

Remove small-sized objects: 5

Remove weakly stained objects: 1

Background Threshold: 40

Post Processing Order: Remove, Merge

Merging Rules: Yes 

Max Combined Area: 400 μm^2^ 

Max Involved Compactness: 0.9 

Group Max: 4 

Min Resulted Compactness: 0.6

**Figure 1:**
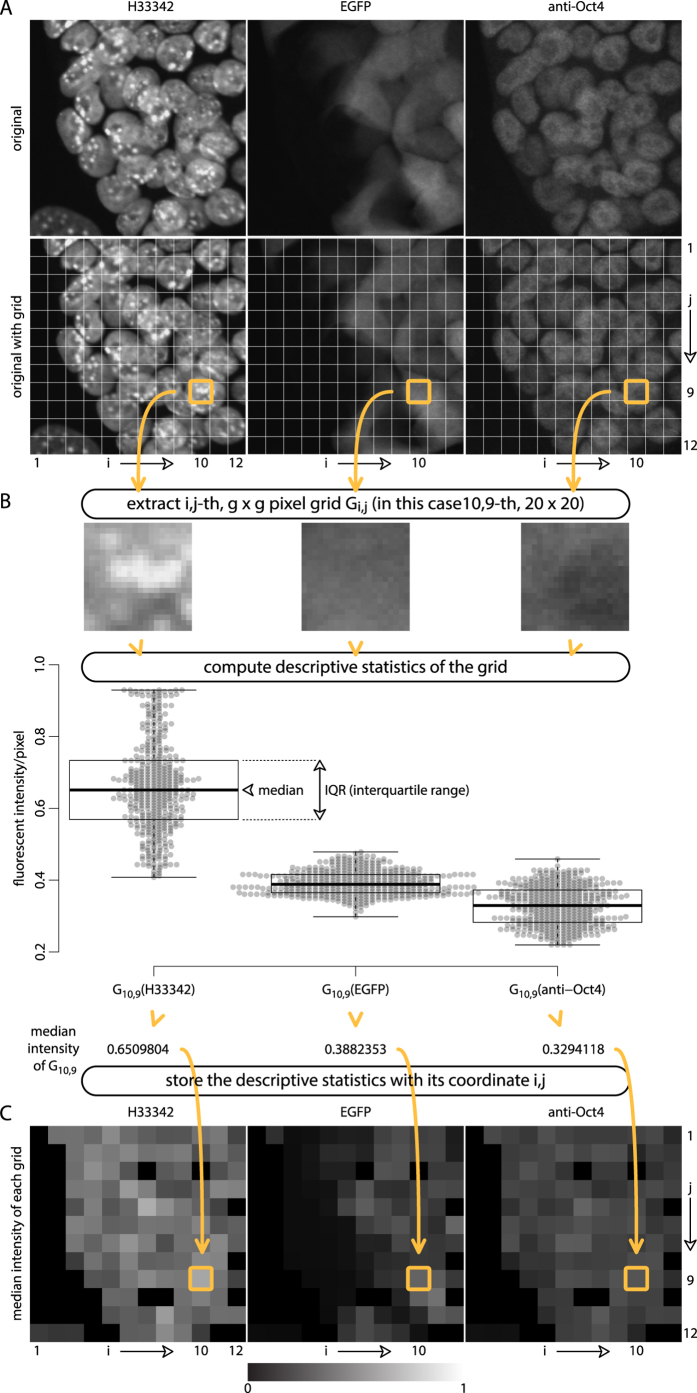
Principle and workflow of GBIQ. (**A**) Three channel test image set (240 × 240 pixel) (upper row) are divided by 20 × 20 (g = 20) pixel grid (lower row) leading 12 × 12 = 144 tiling grids. In this case, *10*,*9*-th grid is indicated (orange square) as an example of following process. (**B**) Magnified images of the *10*,*9*-th grid from each channel (upper row). A set of descriptive statistics of pixel intensity is computed. Boxplots with beeswarms are for quantitative visualization purpose only to aid better understandings to GBIQ, and are not required during the process. (**C**) Median intensity of each grid is stored with its coordinate.

Mean intensities per cell of each channel were used as quantitative dataset. GBIQ was written in R statistics language^4^ and sample scripts are freely available via online repository (https://github.com/yo-ninomy/DemoScripts). During following analysis for 240 × 240 pixel test images, g = 20 is used, so total of 12 × 12 = 144 grids were generated from each channel of the test images (Fig. 1A,C). The functions (GridMedian and GridIQR in following cases) could be implemented as follows using R language where “image” is a gray scale image data and “g” is a size of grid (g). To accelerate the process speed, a part of these functions is replaced with a function written in C++ via Rcpp (see https://github.com/yo-ninomy/DemoScripts).


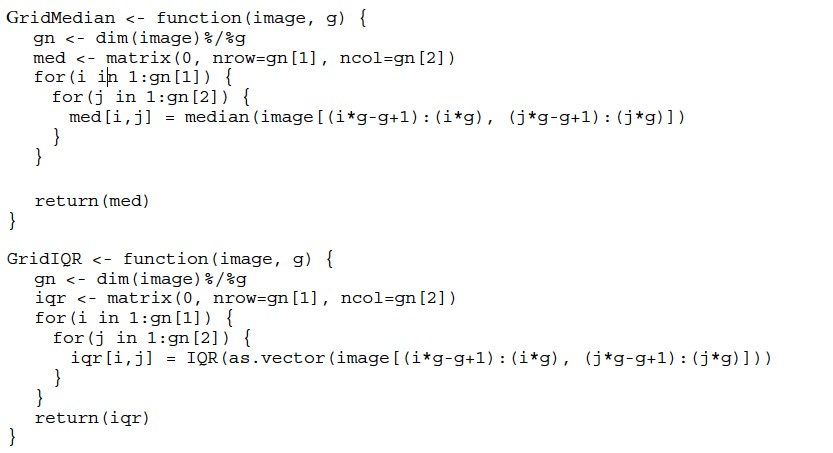


For a classification of a dataset by Gaussian finite mixture model based multivariate clustering fitted by EM algorithm^5^ (Mclust), “mclust” package^6^ was used with its internal BIC (Bayesian information criterion) functionality. Unless otherwise stated, the BIC functionality in the package was used for estimations of the number of cluster (*k*) and the proportion of each cluster. Other than basic R packages and “mclust”, following R packages were used. “EBImage”^7^ for image data I/O, “Rcpp”^8^ for C++ trans-coding, “lawstat”^9^ for Brunner-Munzel Test^10^, “beeswarm”^11^ for beeswarm plot and “plot3D”^12^ for three-dimensional plot. In some cases, *k* was suggested by k-means clustering^13^ using “kselection” package^14^. Analysis by generalized additive models (GAMs) was done using “mgcv” package^15^.

## Results

### Experimental system used in this model case

We chose mESCs to be analyzed since it has been used for many experiments, thus data-rich cell system and easy to manipulate gene expression. We established a permanent cell line harboring PB-EGFP-t2a-Nanos2 gene, by which we can induce EGFP and Nanos2 expression through Dox treatment. Nanos2 is an RNA-binding protein involved in mRNA metabolism during male germ cell development^16^. In our mESC system, the Dox induced Nanos2 expression may affect expression of signature genes for mESCs such as Oct4. The expression level of Nanos2 can be monitored by the intensity of EGFP emission (Pearson’s product-moment correlation indicated reasonably high correlation coefficient r = 0.8508328 using datasets quantified by TQ).

### Workflow of GBIQ

Three channel 8-bit gray-scale fluorescent test images, 240 × 240 pixel each, were captured for EGFP fluorescence and Oct4 expression with H33342 for DNA counterstaining after Dox induction (Fig. 1A). GBIQ application on the small test images showed a nature and tendency of dataset produced by GBIQ. A main function of GBIQ subdivides and tiles an image by a fixed-size (g^2^ pixels) grid (Fig. 1B), computes descriptive statistics (median intensity and interquartile range (IQR) in this article) of each g^2^-pixel grid and stores the statistics with its coordinate (Fig. 1C). Grid size (g) = 20 pixel was used for following GBIQ application on the mESC images and g = 16 was used for images of tissue sections.

Instead of mean intensity, median intensity (Fig. 1B) of each grid was used because its robustness against outliers including noise, debris and artifacts (see Supplementary Fig. S1). Looking at a simulation image created by adding random noise indicates clear difference between median intensity and mean intensity (see Supplementary Fig. S1). As the subtracted image shows greater variances over median intensity (GridMedian), mean intensity (GridMean) is prone to be affected by added noise. An impact of accidentally contaminated debris in an anti-Oct4 fluorescent image is effectively removed by median intensity (GridMedian), not by mean intensity (GridMean), showing another example of its robustness against outliers (see Supplementary Fig. S1, arrowheads). It is the same reason for employing IQR (interquartile range, Fig. 1B), rather than standard deviation, regarding evaluation of intensity variance among pixels within a grid.

**Figure 2:**
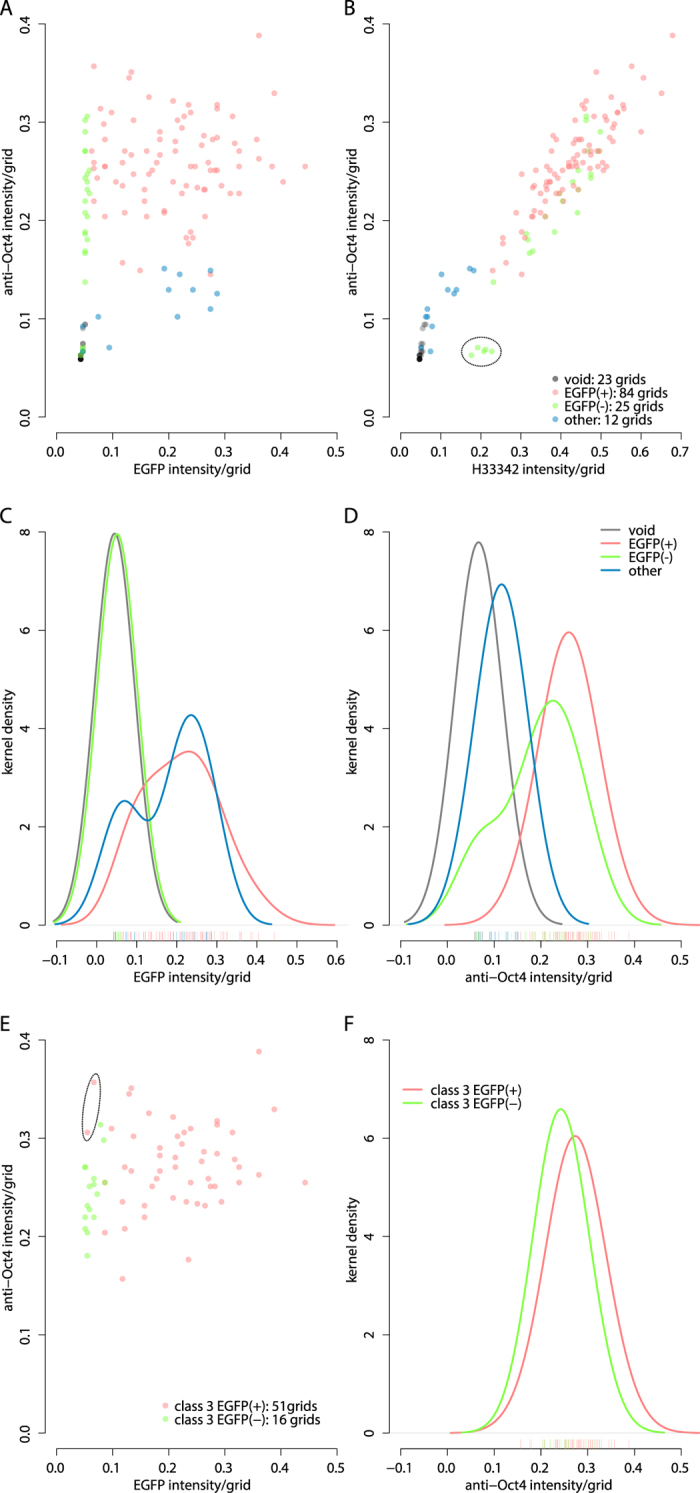
GridMedian extracts features from fluorescent image data while reduce the size of data. (**A,B**) The median intensities of all 144 grids from the test image set (Fig. 1A) are plotted. Anti-Oct4 intensity does not correlate with EGFP intensity (**A**), while well correlate with H33342 intensity (**B**). The 144 grids are classified to 4 sub-classes by Mclust according to the 3-channel intensities (H33342, anti-Oct4 and EGFP) without drawing threshold lines arbitrarily. The 4 sub-classes are shown in 4 different colours and tentatively designated. Misclassified grids are encircled (**B**). (**C**) Kernel density estimations indicate very similar EGFP expression profiles between EGFP(−) and void sub-classes. High-peak of sub-class designated “other” (blue) corresponds cytoplasmic region of EGFP(+) mESCs. (**D**) Oct4 expression profiles between EGFP(−) and EGFP(+) sub-classes suggests higher expression of Oct4 in EGFP(+) mESCs. Smoothing kernel = Gaussian, bandwidth = 0.05. (**E**) After successful application of “Median_IQR filter” (see Supplementary Fig. S2) on the 144 grids, 67 of class 3 grids are again classified by Mclust leading EGFP(+) and EGFP(−) sub-classes. Suspected misclassified grids are encircled. (**F**) The Oct4 expression profiles between 51 of EGFP(+) and 16 of EGFP(−) sub-classes indicate higher expression of Oct4 in mESCs. Sample script (GBIQ_ESC.R) to reproduce the result and figures is available from https://github.com/yo-ninomy/DemoScripts.

**Figure 3:**
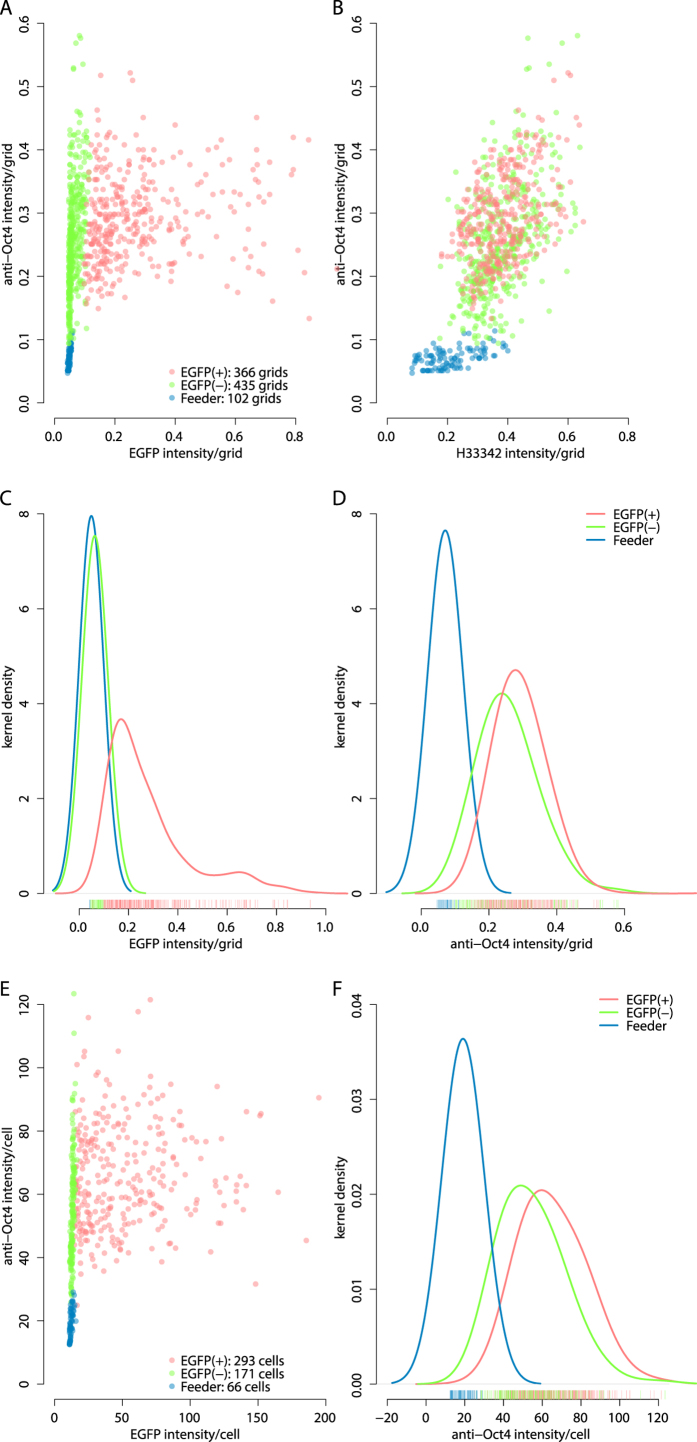
GBIQ reproduces a comparative result to a standard cell-segmentation method. (**A,B**) The 903 observations after applying the “Median_IQR filter” and Mclust on the filtered dataset of 3 full resolution image sets are plotted. (**C,D**) Kernel density estimation of EGFP and Oct4 expression profiles show distinctive properties among the 3 sub-classes (red, green and blue). (**E,F**) Expression level of Oct4 is measured by TQ on the same image sets that consist 3 sets of Dox-treated fluorescent micrographs. From the 3 sets of images, TQ identifies 530 cells that are classified into 3 sub-classes (red, green and blue) by Mclust (**E**). Kernel density estimation shows TQ also reports higher expression of Oct4 in EGFP(+) mESCs (**F**). Smoothing kernel = Gaussian. (**C,D**) GBIQ, bandwidth = 0.05. (**F**) TQ, bandwidth = 10.

**Figure 4:**
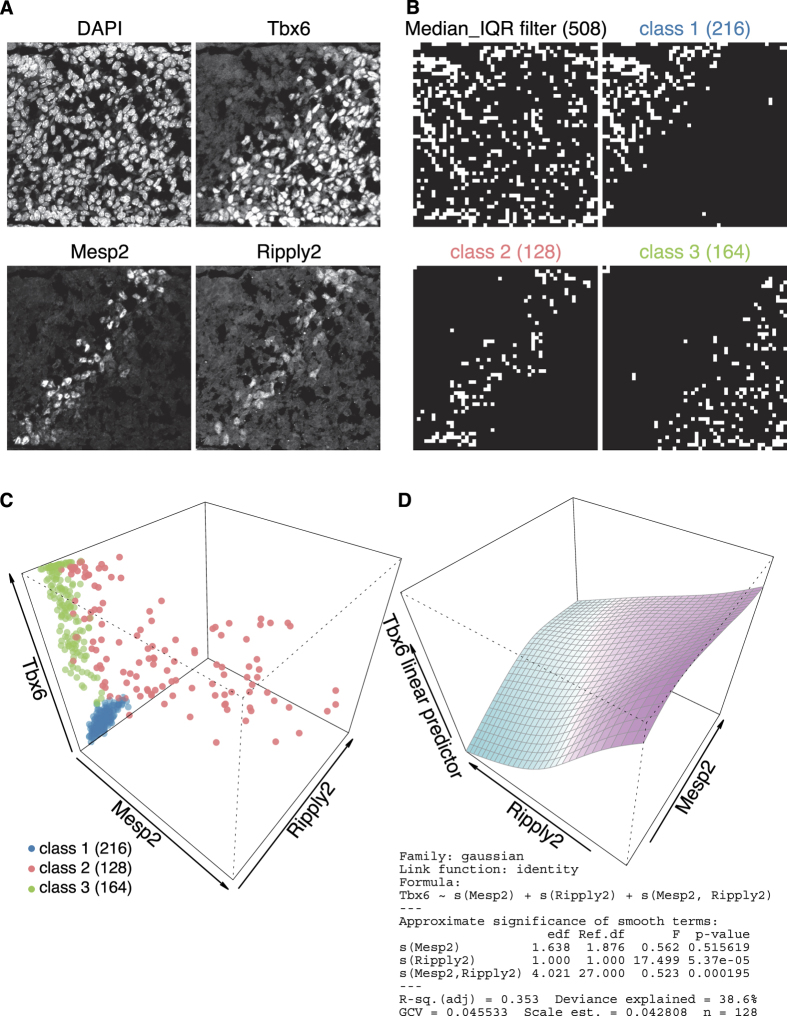
GBIQ application on tissue section uncovers distinctive function of Ripply2. (**A**) Triple immunofluorescent staining of developing tailbud from mouse embryo shows very similar expression patterns of Mesp2 and Ripply2. Nuclear counterstaining by DAPI indicates a high density of mesodermal cells in PSM. Note that expression of Tbx6 is curtailed coinciding with Mesp2 and Ripply2 expression. Top-left towards anterior while bottom-right towards posterior of the embryo, so the A-P axis is perpendicular to stripes of Mesp2 and Ripply2 expression. Each image is 800 × 800 pixel. (**B,C**) These immunofluorescent images (**A**) are processed by GBIQ with g = 16. Applying the “Median_IQR filter” onto DAPI channel extracts reliable 508 observations (**B**, Median_IQR filter, white grids). Further applying Mclust utilizing the 3 factors (median intensities of Tbx6/Mesp2/Ripply2) onto the 508 dataset classifies 3 classes (**C**), which illustrate distinctive tissue architecture (**B**, class1, 2 and 3, white grids). (**C**) All 3 factors are expressed in the class 2 (red), while neither Mesp2 nor Ripply2 expression is evident in the class 1 (blue) and class 3 (green). The expression of Tbx6 is higher in the class 3 (green) than the class 1 (blue). (**D**) GAM analysis of the class 2 dataset indicates negative correlation between Ripply2 and Tbx6. Summary output of the analysis is also shown. Sample script (GBIQ_PSM.R) to reproduce the result and figures is available from https://github.com/yo-ninomy/DemoScripts.

After applying the functions onto each channel, the dataset should be classified to extract clusters of grids (classes) that contain biologically meaningful features. For the classification, Gaussian finite mixture model based multivariate clustering (Mclust)^5^ was applied on the dataset by utilizing 3 explanatory variables (median intensities of the 3 channels, Fig. 1C). Number of clusters (*k*) and its likeliest proportion were determined by Mclust’s internal BIC functionality^6^. The clustering method non-arbitrary classifies the 144 grids from the 240 × 240 pixel test image set (Fig. 1A) to 4 classes (*k* = 4, Fig. 2A,B, red, green, blue and gray) including supposedly 25 of EGFP(−) grids (green) and 84 of EGFP(+) grids (red). The latter shows gradient intensity of EGFP indicating various expression levels of EGFP among the mESCs (Fig. 2A). The rest of 35 grids might be composed with void (cell free region, gray, 23 grids), feeder cells and cytoplasm of the mESCs (blue, 12 grids). Kernel density estimation^17^ of each class (Fig. 2C,D) suggests that Oct4 expression level among EGFP(+) mESCs (red) is higher than that of EGFP(−) mESCs (green, Fig. 2D). But this result should be taken cautiously because of inevitable contamination of non-mESC components and unreliably allocated grids that could lead misclassification. In fact, 5 of EGFP(−) classified grids (Fig. 2B, encircled dotted line) are actually come from a nucleus of feeder cell, not from mESCs (data not shown). Elimination of this class of grids from a dataset is crucial, and requires layers of non-arbitrary classification filters. To this end, variance based filter was introduced.

### Variance based filter for a selection of grids

Few grids whose IQR of H33342 channel is below a certain level should be selected for further analysis. An assumption behind the selection is that if entire pixels of a grid belong to a same cellular domain, for instance cell nucleus, the IQR of the grid should be small since relatively uniform intensity among the pixels (see Supplementary Fig. S2, upper orange square). This also should be applicable if a grid is allocated to void (see Supplementary Fig. S2, upper white square). On the other hand, when a grid contains more than 2 cellular domains, for instance cell nucleus and cytoplasm/void, then the IQR of the grid should increase since large variance of the intensity (see Supplementary Fig. S2, lower white and lower orange squares). In other words, a selection of grids whose median intensity of H33342 is relatively high as well as small IQR is the group of grids that almost exclusively belong to cell nucleus region. Bearing this assumption in mind, an application of Mclust utilizing both median intensity and IQR of the H33342 channel successfully classified the 144 grids into 4 classes (*k* = 4, see Supplementary Fig. S2). A closer look of each class reveals that the “Median_IQR filter” reasonably well performs to classify the grids. For instance, all of class 1 grids belong to void (see Supplementary Fig. S2, gray), while majority of class 3 grids are allocated middle of cell nucleus of mESCs (see Supplementary Fig. S2, red). Boxplots of 4 representative grids from each class confirm that a population of 67 grids with relatively high median intensity and small IQR (class 3 in this case) is the class should be selected for further analysis (see Supplementary Fig. S2).

### Grid size affects performance of classification by “Median_IQR filter”

Needless to say, the size of grid (g) influences on efficiency and performance of “Median_IQR filter”. To see the influence and to establish a good balance between the size and the performance, various sizes of grid, ranging from g = 8 to g = 40 (see Supplementary Fig. S3), were applied on the test H33342 image, then “Median_IQR filter” was performed for a classification of the grids. The filter-selected class or classes should fulfill both 1) mean intensity of grids in the class is greater than overall mean intensity (see Supplementary Figs S2 and S3, vertical lines) and 2) mean IQR of grids in the class is smaller than overall mean IQR (see Supplementary Figs S2 and S3, horizontal lines) criteria (see Supplementary Figs S2 and S3, gray-shaded quadrants). With these criteria, at least 1 class is classified as the selected class in each case of g (see Supplementary Fig. S3), two classes are selected in the case of g = 12 (see Supplementary Fig. S3). A notable exception is the case of g = 30 where no class fulfills the criteria, so a class indicating its mean of median intensity is above the overall mean intensity (see Supplementary Fig. S3) is selected. The 20 × 20 pixel grid size (g = 20) is confirmed as optimal for the test images. Firstly, this size gives highest mean intensity of the filter-selected class (see Supplementary Fig. S2, class 3) over other sizes of grid (see Supplementary Fig. S3). Secondary, the number of grid in the selected class (67) is less than twice of the actual number of nucleus in the H33342 test image (41), which was obtained by manual counting (see Supplementary Fig. S3). Ideally, each nucleus should allocate a grid in the middle, but this situation would hardly happen due to simple tiling algorithm of GBIQ. More than twice of the actual cell number means the size of grid would be too small allowing some nuclei allocate more than 1 grid. Obviously, grid size less than 20 should be unsuitable for the test image as they tend to do multiple sampling to a nucleus in the image (see Supplementary Fig. S3). On the other hand, g = 30 and g = 40 would be too large leading only a few grids are allocated in the middle of nuclei and not producing reliable datasets (see Supplementary Fig. S3). Opting out both less than 20 and more than 24, either g = 20 and g = 24 would be optimal size of grid, g = 20 is chosen based on its higher mean intensity and higher number of grid (see Supplementary Fig. S3) in the filter-selected class.

### GBIQ is an alternative to cell segmentation based image quantification

The 67 of class 3 classified grids are mixed populations composed with EGFP(+) and EGFP(−) grids. Therefore, the class 3 grids were again classified by Mclust utilizing the 3 explanatory variables and automatically assigned two sub-classes; 16 of EGFP(−) grids and 51 of EGFP(+) grids (Fig. 2E). Kernel density estimation of anti-Oct4 median intensity for both sub-classes clearly demonstrates a little (× 1.126546 over EGFP(−)) but firm (Brunner-Munzel Test^10^ reports *p*-value = 0.006064) up-regulation of Oct4 expression in EGFP(+) mESCs (Fig. 2F). To see whether this should be a genuine phenomenon, 3 sets of full resolution (1024 × 1024 pixel) images (see Supplementary Fig. S4) were analyzed using GBIQ. Application of GBIQ onto the 3 image sets produces a large data-matrix of 4 measured variables (median intensities of the 3 channels and IQR of H33342 channel) × 7803 (51 × 51 × 3) observations. A high-content nature of the dataset would make the clustering difficult, an application of the “Median_IQR filter” described above reduces the 7803 observations dataset to reliable 903 observations dataset successfully.

Mclust utilizing the 3 explanatory variables can further classify the 903-observations to 2 sub-classes (red and green, Fig. 3A,B) that resemble the 2 sub-classes of the 67 grids (Fig. 2) classified. Among 366 of EGFP(+) grids in the 903-observations, there are various expression levels of EGFP (red, Fig. 3A,C), and kernel density estimation indicates the EGFP(+) mESCs (red, Fig. 3D) express Oct4 approximately 17% (× 1.168861 over EGFP(−)) higher than EGFP(−) mESCs (green, Fig. 3D). The difference is statistically significant as Brunner-Munzel Test reports *p*-value = 7.216e-13 between 366 of EGFP(+) and 435 of EGFP(−) mESCs regarding anti-Oct4 intensity. H33342 DNA counter-staining intensities between the EGFP(+) and EGFP(−) populations are not statistically significant (see Supplementary Fig. S5, Brunner-Munzel Test reports *p*-value = 0.4135).

To examine whether GBIQ can be a practical alternative to the standard cell-segmentation based image quantification, the 3 sets of images, which have been measured by GBIQ above, were analyzed using a cell-segmentation based fluorescent cytometry (TQ) then both outcomes were compared. TQ identifies 530 cells in the 3 sets of images including 293 of EGFP(+), 171 of EGFP(−) mESCs and 66 of feeder cells (Fig. 3E) that were classified by Mclust utilizing the 3 explanatory variables (mean intensity per cell segments of the 3 channels). Kernel density estimation of the data obtained through either GBIQ (Fig. 3D) or TQ (Fig. 3F) show nearly identical fluorescent intensity profiles. While, as expected, H33342 intensities indicate no difference between the EGFP(+) and EGFP(−) populations (see Supplementary Fig. S5), a marked difference is observed regarding EGFP expression between the populations (Fig. 3C and see Supplementary Fig. S5). TQ also indicates that EGFP(+) mESCs express Oct4 approximately 22% (× 1.218115 over EGFP(−)) higher than EGFP(−) mESCs. These results strongly indicate that GBIQ can draw information paralleled to a cell segmentation based method.

### Application of GBIQ onto tissue sections uncovers hidden gene regulatory network

Histology of tissue sections provides valuable cellular phenotypic information together with anatomical and organ architectural features. Combining the features with quantitative measures would therefore greatly enhance the detective power to uncover previously unseen gene regulatory networks. To show such capability, GBIQ was applied onto immunofluorescent images acquired from tissue sections of mouse embryonic tailbud. While mouse embryo develops, somite, iterative segmental structure of mesoderm, forms from presomitic mesoderm (PSM) along with A-P axis in every 2 hours. During the somitogenesis, anterior limit of a T-box transcription factor Tbx6 expression in PSM (Fig. 4A Tbx6) defines prospective somite boundary, and other mesodermal factors Mesp2 and Ripply2, which express in the vicinity of the prospective boundary (Fig. 4A Mesp2 and Ripply2), are thought to contribute for the degradation of Tbx6^18^. Aim of this image analysis is, from a set of tissue sections, to draw the network that clarifies which molecular entity, either Mesp2 or Ripply2, or both, actually degrades Tbx6. All image datasets of the tailbud analyzed here were part of already published study^3^, so following results are for demonstration purpose only.

By following previously described criteria, g = 16 was chosen and with the grid size, applying “Median_IQR filter” onto DAPI channel sieved reliable 508 observations out of total 2500 (50 × 50) observations (Fig. 4B Median_IQR filter). Based on their Tbx6/Mesp2/Ripply2 expression levels, Mclust successfully classifies the 508 observations to 3 classes (*k* = 3, Fig. 4B), which k-means clustering^13^ suggested. Indeed, these 3 classes (Fig. 4B class 1 to 3) correspond well to intuitive classification and tissue architectural features (Fig. 4A). For instance in the class 2, where all 3 molecular entities express (Fig. 4C red), location of the class 2 observations (Fig. 4B class 2) roughly coincides with stripes of Mesp2 and Ripply2 expression (Fig. 4A Mesp2 and Ripply2).

Utilizing the class 2 observations, a GAM analysis unequivocally predicts that Ripply2 mediates the degradation of Tbx6 (Fig. 4D Ripply2 axis, *p*-value = 5.37e-5). The model also predicts that Mesp2 is unlikely contributor to Tbx6 degradation (Fig. 4D Mesp2 axis, *p*-value = 0.516). By applying the similar workflow on several other immunofluorescent image sets, nearly identical results were repeatedly obtained (see Supplementary Fig. S6). Even though both Mesp2 and Ripply2 expression are confined in a very similar manner (Fig. 4A and Supplementary Fig. S6 Mesp2 and Ripply2), GBIQ successfully represents a reliable dataset that can draw unseen difference between Mesp2 and Ripply2 in terms of a gene regulatory network for Tbx6 degradation in PSM.

## Discussion

Quantitative evaluation of image datasets becomes increasingly common practice in modern biology. Ideally, the evaluation requires non-arbitrary, non-biased measurement of the datasets during initial steps to obtain reproducible quantitative outcome. This leads a demand for a method that is simple enough so it does not need much human intervenes. GBIQ is developed the simplicity in mind and basically requires only one parameter, the size of grid (g), to set. Traditionally, cell-segmentation based image processing has been the standard method to analyze microscopy image datasets. This method can eliminate much of noise and unwanted artifacts by setting a number of parameters during the process. On the other hand, the number of parameters to be set by an operator means that the number of arbitrary and biased interventions should inevitably be introduced to the outcome. In addition, the interventions would prevent an efficient implementation of the method to a fully automated process for biological imagings. Its parameter-free design allows GBIQ to be easily implemented into the automated processing.

The size of grid (g) depends on magnification and resolution of the images to be processed, as well as properties of the target entities to be measured. For nuclear localized molecular entities, systemic explorations agreed that around 4 grids covering an entire cell nucleus would be an optimal size of the grid leading a well balance between the reducing noise and dimensions, and the retaining features. Recently, a machine learning based biological image classifier reported a parameter, called Calibration Factor, to achieve optimal classification outcome^19^. The factor is based on optical magnification, image sensor’s resolution and binning coefficient to identify target biological objects. These 3 determinants essentially mean that the factor depends on relative size of target objects in an image dataset to be processed. The size of grid was set to either g = 16 (for tissue sections) or g = 20 (for *in vitro* mESCs) in this study, but this could be easily altered if target entities are localized sub-cellularly or need to be analyzed at tissue level rather than cellular level, in a similar manner that Calibration Factor was determined. In other words, the both systems should be told what types of entities to be analyzed before actually commence to analyze them. Once the grid size has been set, a pile of images can be processed in a way that requires no further parameter modification.

Certainly the nature of GBIQ has a plenty of rooms to contain errors and noises in the quantitative outcome. Employing an array of classification/clustering methods as a part of post-process for the outcome can address this problem. To simplify the process flow, Gaussian finite mixture model was entirely adopted to classify the dataset in this article. Even though the clustering method sometimes misclassified a few grids (Fig. 2E, encircling dotted line and Supplemental Fig. S6, class 3 red-coloured grids), the overall classification result clearly indicates biologically meaningful sub-classes. To improve the classification efficiency, other machine learning algorithms such as Decision Trees, Random Forest, Support Vector Machine and Neural Networks, can also be implemented as post-processes. These algorithms might be useful to reduce the errors and noises that arise due to simple tiling of the grids. If iterative feedback fitting of these algorithms can pinpoint grids on specific cellular domain, for instance cell nucleus, GBIQ could become more target-feature oriented image detection and quantification package. But the implementations would be complex and beyond the scope of this article. The current form of GBIQ produces a large matrix of data that can be classified to few biologically meaningful sub-classes without arbitrary thresholds and biases.

As any method comes with its own advantages and disadvantages, GBIQ and cell-segmentation based methods are no exception. Obviously, GBIQ cannot quantify cellular morphometrics; another phenotypical and valuable information that should be measured by cell segmentation-based methods. The current version of GBIQ focuses on fluorescent intensity rather than cell morphology, because of its primary motivation is abandoning cell identification process from a quantification workflow for the sake of simplicity and minimizing human interventions. Without cell segmentation, the cellular morphometrics cannot be performed. Regarding the fluorescent intensity, segmentation-based image quantification is still preferable whenever the subject molecular entities distribute different domains of a cell. Thus, if a marker entity of particular cell type localizes cell nucleus and a molecular entity in question distributes cytoplasm, or vice versa, GBIQ cannot deal with that situation, the cell-segmentation should be only viable option to quantify and analyze the entities.

On the other hand, if the entities distribute same domain of a cell (e.g. Oct4/EGFP in cell nucleus of mESCs and nuclear localization of Tbx6/Meap2/Ripply2 in PSM), GBIQ performs well as much as cell-segmentation based method would perform (Fig. 3) without comprehensive optimization of parameters. GBIQ even outperforms the cell-segmentation if cell/tissue density of the subject is very high in which cell-segmentation is virtually inapplicable. In fact, GBIQ was successfully employed to elucidate a relationship among Tbx6, Mesp2 and Ripply2, all of the 3 factors are nuclear protein, in mouse mesoderm during somitogenesis^3^. Because cell density of the mesoderm is very high, cell segmentation could not be applied on the image datasets, GBIQ was only practical solution for the quantification. Indeed, GBIQ streamlined the image analysis workflow in a semi-automated manner, processed a pile of image datasets and illustrated relativities among the 3 factors quantitatively as exemplified in this article (Fig. 4 and Supplementary Fig. S6). These examples consistently suggest that Ripply2, not Mesp2, is the direct contributor to the degradation of Tbx6, and this was indeed proven by genetic and biochemical studies^3^.

In conclusion, GBIQ should be a comparable alternative to the cell segmentation based image quantification where certain conditions are appropriate. Its parameters minimization design allows GBIQ to be readily implemented in an automated workflow for non-arbitrary and non-biased quantification of fluorescent images. GBIQ would also be applicable where cell density of subjects is high such as tissue section, onto which conventional cell-segmentation based methods are difficult to apply, leading a new way to move forward the data-centric analysis of biological systems.

## Additional Information

**How to cite this article**: Ninomiya, Y. *et al*. GBIQ: a non-arbitrary, non-biased method for quantification of fluorescent images. *Sci. Rep*. **6**, 26454; doi: 10.1038/srep26454 (2016).

## Supplementary Material

Supplementary Information
