# Supplementary material for: GBIQ: a non-arbitrary, non-biased method for quantification of fluorescent images
Source: Sci Rep. 2016 May 23;6:26454. doi: 10.1038/srep26454 (PMC4876397; doi:10.1038/srep26454)
Supplement: Supplementary Information [file srep26454-s1.pdf]

Supplementary Figures

GBIQ: a non-arbitrary, non-biased method for quantification of fluorescent images

Youichirou Ninomiya, Wei Zhao and Yumiko Saga

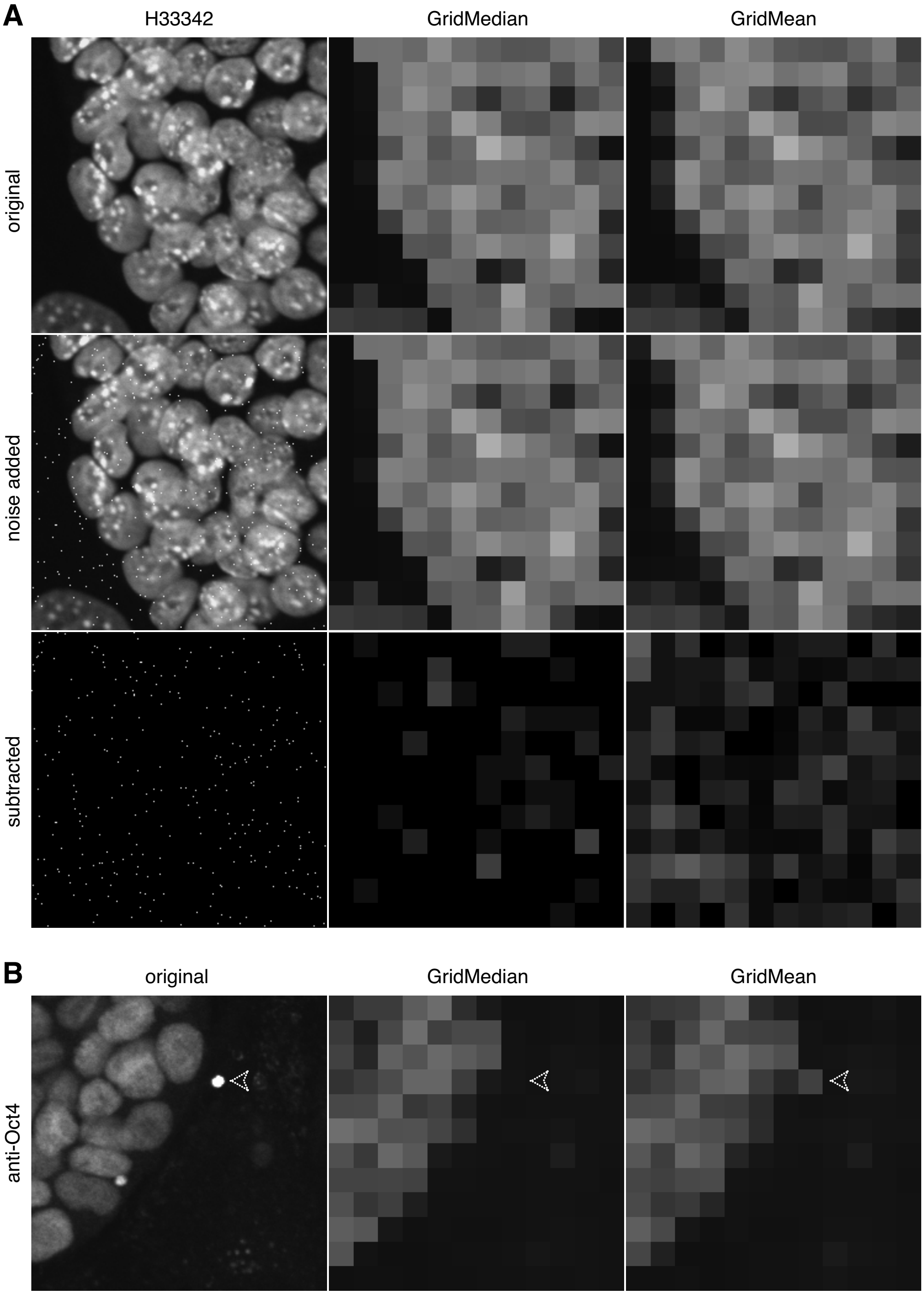

Supplemental Figure S1. Median intensity is preferable over mean intensity.

(A) Robustness of median intensity against noise. A DNA counterstained image (Original H33342) is artificially contaminated with randomly distributed bright spot (Noise added H33342), then both images are processed either GridMedian or GridMean function (top and middle rows). GridMean is a derivative function from GridMedian, in which mean replaces median. Subtracted images (|imageNoise added - imageOriginal|) indicate differences between original and noise-contaminated images (bottom row). In cases of GridMedian and GridMean, the differences are enhanced 30 times to illustrate a robustness of median intensity as descriptive statistics of grids. (B) A real-life example of noise and debris elimination by applying GridMedian function. Arrowheads indicate the location of a debris. While the debris affects mean intensity (GridMean), median intensity efficiently eliminates the debris (GridMedian).

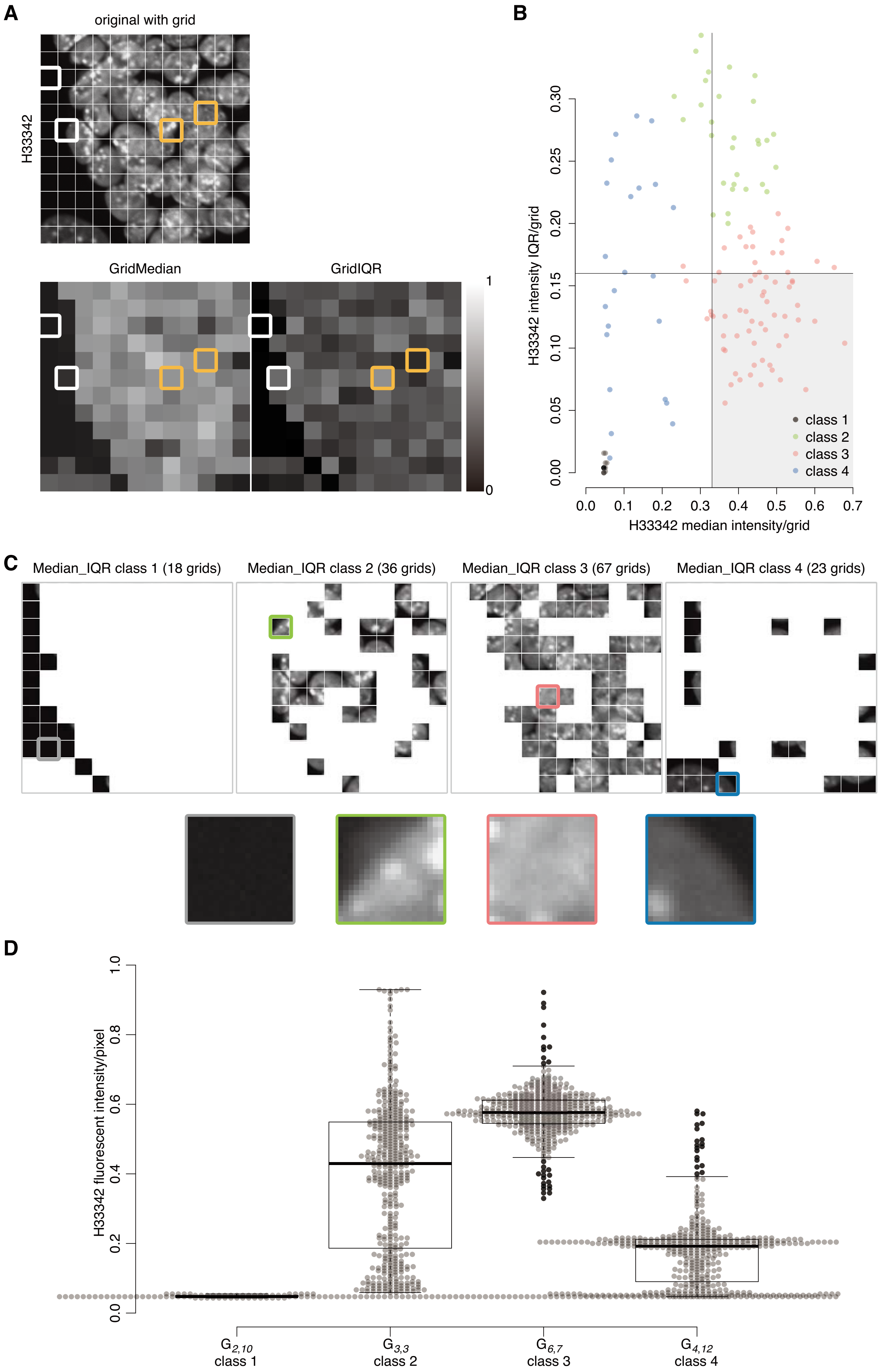
A
original with grid
H33342
GridMedian
GridIQR
1
0
B
H33342 intensity IQR/grid
H33342 median intensity/grid
0.00
0.05
0.10
0.15
0.20
0.25
0.30
0.0
0.1
0.2
0.3
0.4
0.5
0.6
0.7
class 1
class 2
class 3
class 4
C
Median_IQR class 1 (18 grids)
Median_IQR class 2 (36 grids)
Median_IQR class 3 (67 grids)
Median_IQR class 4 (23 grids)
D
H33342 fluorescent intensity/pixel
0.0
0.2
0.4
0.6
0.8
1.0
$G_{2,10}$
class 1
$G_{3,3}$
class 2
$G_{6,7}$
class 3
$G_{4,12}$
class 4

Supplemental Figure S2. Selection of reliable grids by applying "Median_IQR filter".

(A) H33342 test image is processed by GridMedian and GridIQR functions. Orange and white coloured squares in GridMedian indicate pairs of grids whose median intensities are at similar level. These girds however show different level of IQR. (B) Utilizing the median intensity and IQR of grids, Mclust classifies the 144 grids into 4 sub-classes (class 1 to 4). (C) Mapping each class onto original H33342 test image reveals that a group of grids belong to same class have similar properties. (D) Each class representative grid quantitatively shows distinctive properties of image.

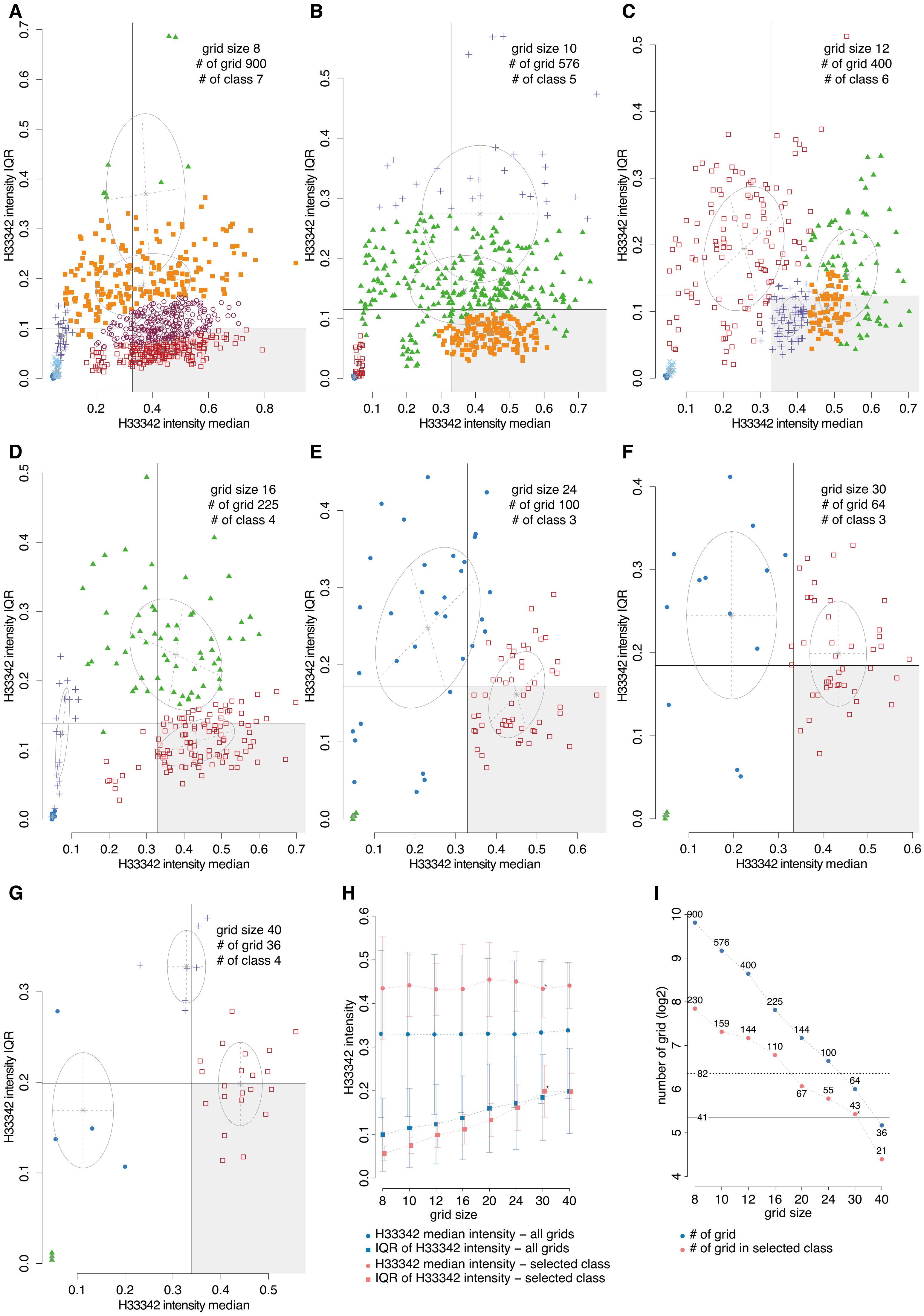
A
grid size 8
# of grid 900
# of class 7
B
grid size 10
# of grid 576
# of class 5
C
grid size 12
# of grid 400
# of class 6
D
grid size 16
# of grid 225
# of class 4
E
grid size 24
# of grid 100
# of class 3
F
grid size 30
# of grid 64
# of class 3
G
grid size 40
# of grid 36
# of class 4
H33342 intensity IQR
H33342 intensity median
H
H33342 intensity
grid size
H33342 median intensity – all grids
IQR of H33342 intensity – all grids
H33342 median intensity – selected class
IQR of H33342 intensity – selected class
I
number of grid (log2)
grid size
# of grid
# of grid in selected class

Supplemental Figure S3. Size of grid (g) affects classification efficiency.

(A-G) GBIQ with various sizes of grid, ranging from 8 to 40, were applied on the H33342 test image (Fig. 1A), then the classification by “Median_IQR filter” criteria (gray shaded area, see Results for details) was carried out. Scattered-plots with symbols in different colours and shapes show classes classified by the filter. Solid ovals and dashed lines represent estimated proportion of Gaussian distribution to the classes. Vertical and horizontal lines indicate overall mean values of median intensity and IQR of all grids respectively. (H) Mean values of H33342 median intensity (circles) and IQR (squares) for both all grids (blue) and the filter-selected classes (red) show successful selection of reliable grids. Large grid size tends to give large IQR (blue squares). The filter-selected classes always have higher mean value of median intensity (red circles) than overall mean (blue circles), and lower mean value of IQR (red squares) than overall mean (blue squares) except in the case of g=30 (asterisks). At g=20, the filter-selected class (see Supplementary Fig. S2B, class 3) gives highest mean value of median intensity. (I) As size of grid is getting larger, total number of grid (blue) as well as number of grid in the filter-selected class (red) is getting smaller. Since actual number of cell nucleus in the H33342 test image (Fig. 1A) is 41, the number of grid in the filter-selected class should be in between 41 and 82 (see Results for details). Sample script (GBIQ_GridSize.R) to aid for selecting optimal size of grid and to reproduce figures H and I is available from https://github.com/yo-ninomy/DemoScripts.

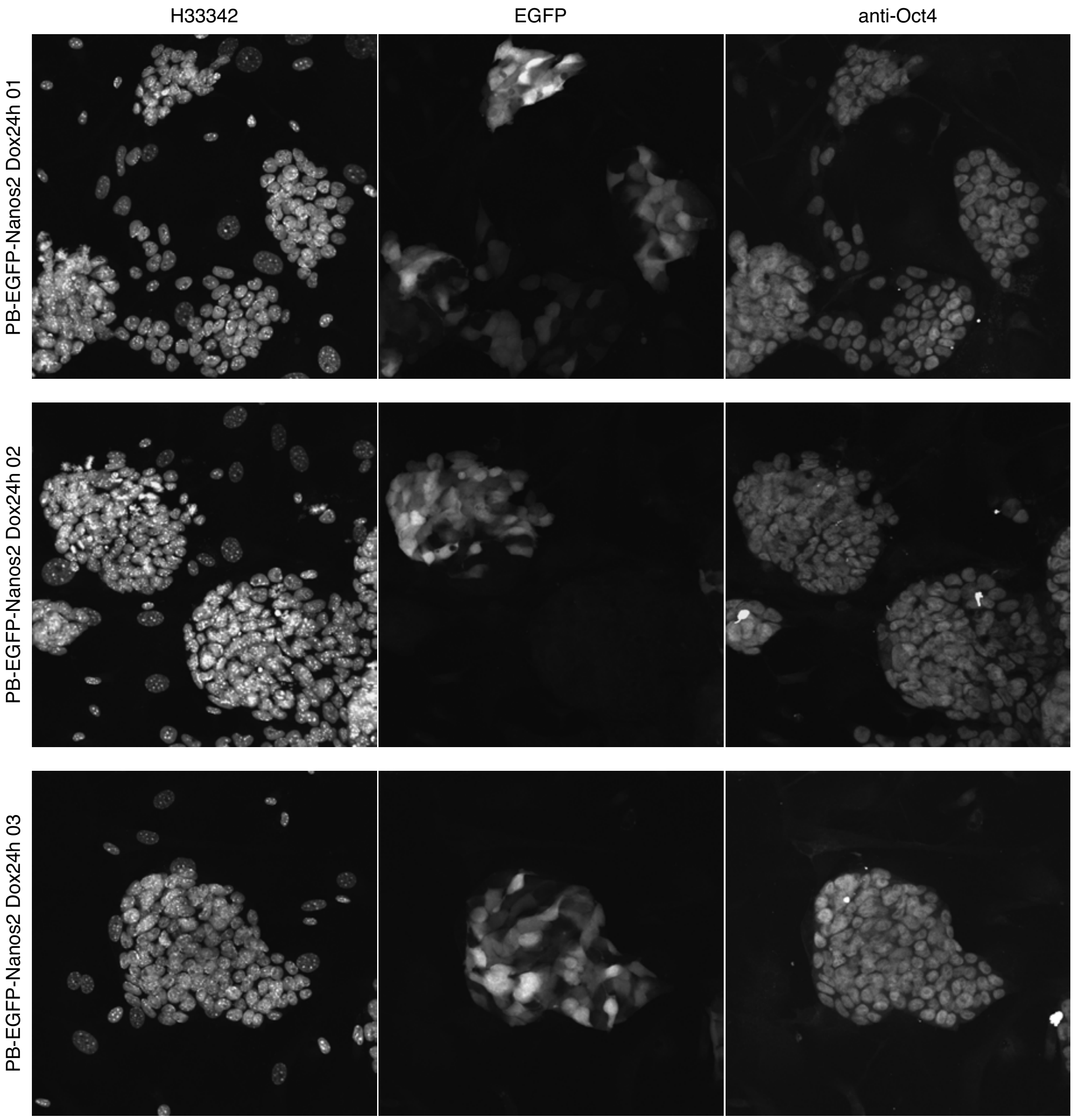

Supplemental Figure S4 Full resolution image sets to be analyzed by both GBIQ and TQ

Three sets of full resolution (1024 x 1024 pixel) images of H33342, EGFP and anti-Oct4 channels acquired from PB-EGFP-Nanos2 mESCs treated with Dox for 24 hours (see Materials and Methods for details). Results of application of both GBIQ and TQ onto these sets are shown in Fig. 3 and Supplementary Fig. S5.

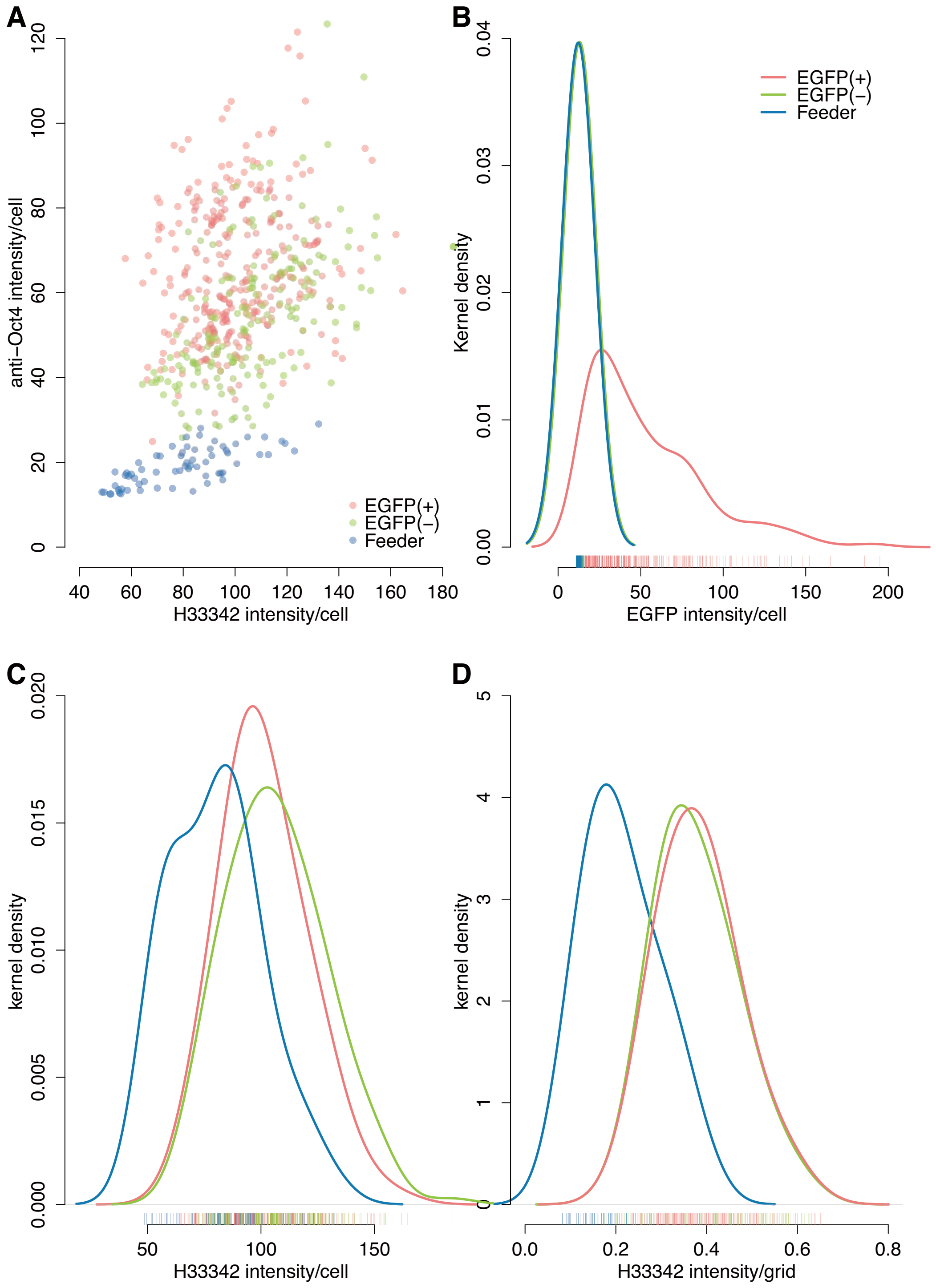

Supplemental Figure S5. TQ also produces very comparable dataset to GBIQ.

TQ reveals differences of expression profiles among EGFP(+) and EGFP(-) mESCs, and Feeder cells (A and B). These are counterparts of Fig. 3B and 3C respectively indicating both GBIQ and TQ reproduce resembling results from same image sets. Between EGFP(+) and EGFP(-) mESCs, H33342 profiles are almost identical and this could be illustrated by TQ (C) and GBIQ (D) in very similar fashion. Smoothing kernel=Gaussian. B and C: TQ, bandwidth=10. D: GBIQ, bandwidth=0.05.

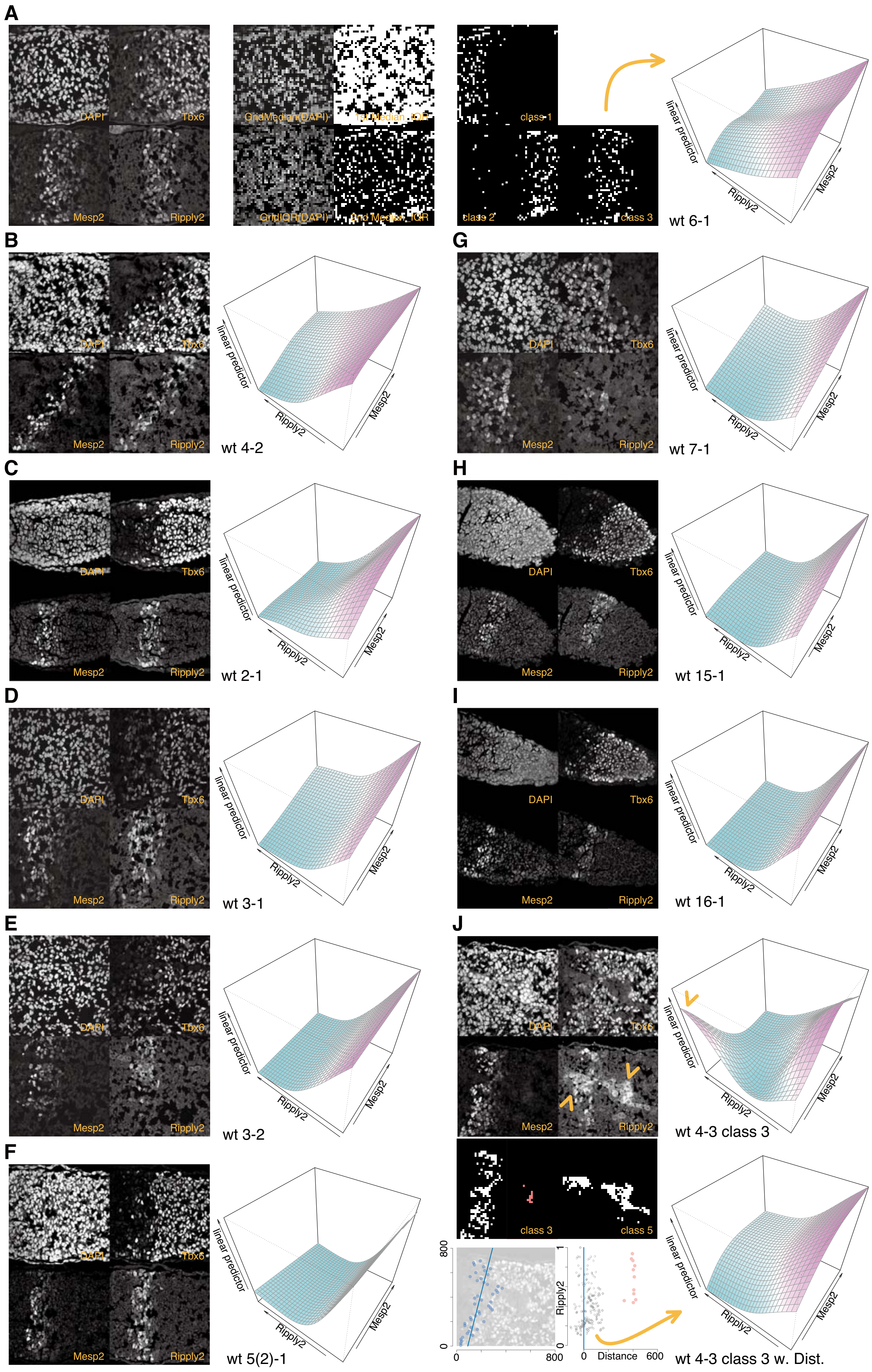
A
DAPI
Tbx6
Mesp2
Ripply2
GridMedian(DAPI)
1st Median_IQR
GridIQR(DAPI)
2nd Median_IQR
class 1
class 2
class 3
linear predictor
Ripply2
Mesp2
wt 6-1
B
wt 4-2
C
wt 2-1
D
wt 3-1
E
wt 3-2
F
wt 5(2)-1
G
wt 7-1
H
wt 15-1
I
wt 16-1
J
wt 4-3 class 3
class 3
class 5
800
0
800
1
Ripply2
0
0
Distance
600
wt 4-3 class 3 w. Dist.

Supplemental Figure S6. GBIQ workflow consistently elucidates insight into gene regulatory network from tissue sections.

(A-J) All of tissue sections were prepared from PSM of wild-type (wt) mouse embryos. (A) Two layers of "Median_IQR filter" effectively select reliable grids. GridMedian(DAPI) and GridIQR(DAPI) are used for selection of grids that are allocated in the middle of cell nucleus. The first layer of the filter segregates nuclear region and void that indicates near zero median intensity and very narrow IQR (1st Median_IQR), then the second layer of the filter selects "high median intensity and narrow IQR" grids (2nd Median_IQR). Mclust utilizing the grid intensities of Tbx6, Mesp2 and Ripply2 classifies the filtered grids to 3 classes (class 1-3). GAM analysis using the class 3 dataset reports negative correlation between Tbx6 and Ripply2. g=16. (B-I) The workflow described above has been applied on following 8 different image sets to elucidate a gene regulatory network of Tbx6 degradation. Except slight differences of the predictor planes, all of the 8 cases consistently suggest that Riply2 is the major contributor for the degradation of Tbx6 as they report firm negative correlation between Tbx6 and Ripply2. g=16. (J) Due to strong auto-fluorescence from cytoplasm, blood cells in Ripply2 image (Ripply2, orange arrowheads) are classified as class 5, but some of them are misclassified to class 3 (class 3, red-coloured grids). The misclassification causes a flipping of the linear predictor plane towards highest expression of Ripply2 (wt 4-3 class 3, orange arrowhead). To eliminate the flip, a frontline of Tbx6 expression is estimated by Linear Model fitting (blue dots and line) 4, then the class 3 grids in the vicinity of the frontline are selected (class 3, white-coloured grids) based on an assumption that the degradation of Tbx6 takes place in line with both Mesp2 and Ripply2[4]. This additional layer of information regarding the distance between each grid and the frontline (blue lines) successfully reduces the noise and draws a consistent result with other image sets by GAM analysis (wt 4-3 class 3 w. Dist.). g=16.
